# Resilience of Soil Microbial Communities to Metals and Additional Stressors: DNA-Based Approaches for Assessing “Stress-on-Stress” Responses

**DOI:** 10.3390/ijms17060933

**Published:** 2016-06-14

**Authors:** Hamed Azarbad, Cornelis A. M. van Gestel, Maria Niklińska, Ryszard Laskowski, Wilfred F. M. Röling, Nico M. van Straalen

**Affiliations:** 1 Institut National de la Recherche Scientifique-Institut Armand-Frappier, 531 Boulevard des Prairies, Laval, H7V 1B7 QC, Canada; 2 Institute of Environmental Sciences, Jagiellonian University, Gronostajowa 7, 30-387 Krakow, Poland; maria.niklinska@uj.edu.pl (M.N.); ryszard.laskowski@uj.edu.pl (R.L.); 3 Department of Ecological Science, Faculty of Earth and Life Sciences, Vrije Universiteit, de Boelelaan 1085, 1081HV Amsterdam, The Netherlands; kees.van.gestel@vu.nl (C.A.M.v.G.); nico.van.straalen@falw.vu.nl (N.M.v.S.); 4 Department of Molecular Cell Physiology, Faculty of Earth and Life Sciences, Vrije Universiteit, de Boelelaan 1085, 1081HV Amsterdam, The Netherlands; wilfred.roling@vu.nl

**Keywords:** resilience, metal pollution, microbial communities, secondary stressors, resistance, microbial stability

## Abstract

Many microbial ecology studies have demonstrated profound changes in community composition caused by environmental pollution, as well as adaptation processes allowing survival of microbes in polluted ecosystems. Soil microbial communities in polluted areas with a long-term history of contamination have been shown to maintain their function by developing metal-tolerance mechanisms. In the present work, we review recent experiments, with specific emphasis on studies that have been conducted in polluted areas with a long-term history of contamination that also applied DNA-based approaches. We evaluate how the “costs” of adaptation to metals affect the responses of metal-tolerant communities to other stress factors (“stress-on-stress”). We discuss recent studies on the stability of microbial communities, in terms of resistance and resilience to additional stressors, focusing on metal pollution as the initial stress, and discuss possible factors influencing the functional and structural stability of microbial communities towards secondary stressors. There is increasing evidence that the history of environmental conditions and disturbance regimes play central roles in responses of microbial communities towards secondary stressors.

## 1. Introduction

Soil is known as a major sink for metals released into the environment. In many industrialized countries, soil pollution has become a serious problem [1]. Metal processing activities such as mining, smelting, as well as the use of meta-containing compounds (e.g., pesticides), sewage sludge application, and the production of industrial waste can contaminate the environment and cause serious effects on ecosystems. Metals are the most common and widespread pollutants, and due to their toxicity, metal pollution represents a potential risk for organisms living in soil, such as microbial communities [2].

Soil microbial activity has great impact on the functioning of whole ecosystems, as microorganisms are key players in such pivotal ecosystem functions as biogeochemical cycling and the decomposition of dead organic matter [3,4,5,6]. Shifts within the microbial community composition (see Table 1 for definitions of terms) in organic forest soils caused by metal pollution may affect decomposition rates and CO_2_ production [7]. A significant volume of research has been conducted to evaluate the effects of metals on soil organisms [8,9], and particularly on soil microbial communities [10,11]. Several reviews have already provided an excellent overview of the progress that has been made in recent years in our understanding of the stability of microbial communities under secondary stressors, *i.e.*, in “stress-on-stress” situations [12,13,14,15,16]. In the present work, we have primarily focused on deployment of molecular approaches, such as 16S rRNA sequencing and metagenomic studies, with a special focus on long-term polluted soils that tell us about microbial diversity, community membership and functional potentials of microbial communities in the environment.

Giller *et al.* [11] argued that, although there have been significant advancements in the field of soil ecotoxicology, our understanding of effects of long-term metal contamination in the natural field situation is far from satisfactory. They emphasized the need for long-term experiments or field studies that are important for the understanding of chronic exposure to metals [11]. In laboratory studies with metals mixed in with the soil as inorganic salts, toxicity and bioavailability may change over time as a result of ageing and equilibration effects. These processes impede an easy extrapolation of laboratory-based data to natural field conditions. Studies of metal contamination in soil microorganisms have shown that metal-tolerance may be selected within a few weeks or months; however, complete community adaptation to metal pollution may take years to develop. Under field conditions, native soil microbes will probably be well adapted to the prevailing conditions of the particular soil, for example, to the background level(s) of metal(s) or the contaminated level(s) [17,18]. In field-contaminated sites, depending on the level of pollution and exposure time, selection of resistant mutants, including horizontal gene transfer or species sorting, is only evolving slowly. Only the long-term exposure to metal pollution leads to adaptation of the microbial communities that can survive and persist in a metal-polluted environment [19,20]. In this review, we consider studies in polluted areas with a long-term history of contamination.

Metal contaminated environments were shown to exert a high selective pressure towards the transfer of metal-resistance genes, developing thus resistance against metal concentrations [21,22,23]. Recent advances in molecular biology have shed more light on the role of the diversity and functioning of soil microbial communities in metal-polluted environments. Molecular analysis of the diversity and expression of metal-resistance genes is providing a growing body of knowledge of the effects of metal pollution in the field. For example, soil bacteria exposed to long-term arsenic contamination (337 mg·kg^−1^) were shown to acquire a diverse array of arsenite-resistance genes (*ACR3*, *arsB*) by horizontal gene transfer [23]. Antibiotic resistance genes and genes for metal resistance can be linked, if present on the same mobile genetic elements. In that way, metal pollution may also promote antibiotic resistance in microorganisms [24,25]. Several field studies on long-term metal polluted areas have shown shifts in microbial community structure and functions or increased occurrence of resistance genes that have made metal-resistant communities stable under long-term exposure to metal pollution [26,27,28].

In addition to genetic linkage, co-selection of traits is also affected by trade-offs among physiological variables. When exposed to stress, microbes have to stage physiological defence mechanisms, which will require extra energy, with subsequent effects elsewhere in the metabolism. The action of such trade-offs due to different costs of tolerance can be understood through resource partitioning theory [29]. As energy available for life history strategies/processes such as growth, maintenance, and reproduction is limited, and increased resource allocation to one particular trait results from diverting resources from other traits. In metal polluted ecosystems, physiological defence mechanisms, such as conversion of a metal to a less toxic form followed by an efflux, can thus be expected to reduce availability of the energy for growth and reproduction [30].

Metal-contaminated ecosystems may contain strains or species selected for general resistance or tolerance, as a result of competitive exclusion of metal-sensitive strains by metal-resistant strains, and so may be able to cope better with a range of secondary stressors. This could happen when genetic or physiological changes provide resistance against more than one stress factor [31], which has been documented in several studies [32,33,34]. For instance, in microcosms exposed to copper at agronomical concentration (CuCl_2_: 50 mg·kg^−1^), as a primary mild stress, Philippot *et al.* [34], reported a higher resilience of the nitrate reduction rates to mercury stress (HgCl_2_: 100 mg·kg^−1^) as a secondary stress. On the other hand, resistance to metals may make the metal-resistant communities more susceptible to other stress factors [32,35]. This has also been shown in several studies for soils contaminated with such metals as copper, mercury and zinc [33,35,36,37]. Tobor-Kapłon *et al.* [35] found a lower functional resistance of soil microbial communities (decomposition of freshly added lucerne meal) in soils that had been exposed to Cu pollution for more than twenty years upon an additional lead stress (1000 mg·kg^−1^). The most polluted soils contained the least Pb-resistant communities. Based on these contradictory results, it is thus difficult to predict the stability of soil microbial communities in the face of secondary stressors in long-term metal polluted soils.

The main question of this review is why there is such a wide variability in results of studies in which the stability of microbial communities under stress was evaluated. In this review, we focus on the application of advanced molecular techniques, as they have been used as primary means to characterize microbial communities in recent years. We discuss studies on the stability of chronically exposed soil microbial communities, and consider their resistance and resilience, when exposed to additional stressors. We also present suggestions and recommendations for future research on “stress-on-stress” responses of soil microbial communities.

## 2. Stability, Resilience and Tolerance of Microbial Communities

The ability of microbial communities to adapt to and recover from stress factors is defined as microbial stability, as understood in macro-ecology [38,39,40]. Microbial stability comprises both resistance and resilience (see Table 1 for definitions of terms). Stability has many different meanings in the literature. Here “community stability” is defined as the ability of a microbial community to withstand (resistance) or to recover (resilience) from metal pollution. An important point that must be considered is that the stability concept refers to distinct components of the microbial response to stress factors, that is, microbial diversity, community structure and microbial function (e.g., respiration rate and enzyme activities). It is clear is that the terms referring to resistance and tolerance have often been used interchangeably in the literature.

In the context of this review, we address the importance of metal pollution effects on the functional and structural stability of soil microbial communities (the term “microbial community structure” is understood here as the species composition of a microbial community and the abundances of particular species). Contradictory results have been observed in field studies, which will be discussed in more detail in the following section. Some of these studies demonstrate a great sensitivity of microbial communities to metal pollution (e.g., decline of microbial diversity) while others show resistance/tolerance (effects of metals on microbial communities is either negligible or small) or resilience (metal pollution can have transient effects on microbial communities followed by a community adaptation to metal exposure and recovery of the community to a new stable state) towards metal exposure in chronically polluted soils. We consider “community resilience” to describe the ability of a population to survive and persist in polluted areas with a long-term history of contamination [41].

We need to point out that community composition to some extent involves stochastic, non-deterministic processes [42]. Therefore, the definition of resilience needs to include recovery to a new stable state within the normal operating range of the system [43,44]. Care should be taken when trying to draw conclusions about microbial sensitivity or stability towards metal stress. For example, there is a different range of sensitivities of microorganisms; some microbes are fairly sensitive to metals, such as rhizobia [45], while others are insensitive (nitrifiers and microorganisms capable of decomposing organic matter) [46]. Species of microorganisms [47], strains of the same species [48] and also activities of the same microbial species [19] can show substantial differences in their sensitivity to metal stress [49].

## 3. Response of Microbial Communities to Metal–Pollution Stress

Singh *et al.* [2] studied the impact of metal stressors on soil microbial communities using 454 pyrosequencing of 16S rRNA genes and a DNA microassay detecting functional genes (GeoChip 3) in two grassland plots from two long-term (11-year-old) experiments that received annual addition of Cu and Zn contaminated sludge to obtain target concentrations of Cu: 50 and 200 mg·kg^−1^; Zn: 150 and 450 mg·kg^−1^. Their results showed that long-term metal stress resulted in a significant decline in microbial diversity, associated with the loss of some key functional groups with specialized functions. Sheik *et al.* [50] also used pyrosequencing to study the effects of long-term exposure to fairly low As (14 mg·kg^−1^) and Cr (VI) (25 mg·kg^−1^) concentrations on soil microbial communities. They observed a marked reduction in bacterial alpha diversity in soils contaminated for more than 40 years with arsenic and chromium in comparison with control soils. Zhou *et al.* [51] studied the effect of the long-term use of Cu-containing fungicides (256 mg·kg^−1^ in most polluted soils) on bacterial community structure and the diversity of operational taxonomic units (using denaturing gradient gel electrophoresis (DGGE) analysis) in citrus grove soils. Their results indicated that long-term application of Cu-containing fungicides had negative effects on bacterial community diversity (measured as operational taxonomic units (OTUs) diversity based on DGGE) in these soils. Using the same approach, Joynt *et al.* [52] studied the effect of long-term metal (lead and chromium) and organic contamination on microbial community structure. They reported significant reduction in microbial community richness in soils contaminated with both metals (Pb: ranged from 1 to 17,000 mg·kg^−1^ and Cr: from 4 to 3200 mg·kg^−1^) and hydrocarbons in comparison with non-contaminated soil.

In contrast to the above-mentioned studies, other researchers have found that long-term metal exposure leads to changes in the composition of microbial communities and towards a higher level of physiological adaptation [60,61,62,63,64,65]. Some studies have also demonstrated “community tolerance” by maintaining microbial communities under long-term exposure to elevated metal concentrations [66,67].

Gołębiewski *et al.* [68] studied bacterial communities (using pyrosequencing) from five study areas contaminated with Cd (3 to 4 mg·kg^−1^), Pb (203 to 1378 mg·kg^−1^), Zn (749 to 2000 mg·k^−1^) and Cr (100 to 760 mg·kg^−1^) in the vicinity of a lead and zinc ore enrichment facility and a chromium green-producing facility in Southern Poland. They found that, in spite of the very high Pb and Zn concentrations in soil, the abundance of soil bacterial communities at the level of phyla, the species richness, diversity, and evenness were similar in all samples, irrespective of the contamination level. Berg *et al.* [69] used tag-coded pyrosequencing of the 16S rRNA gene to evaluate the impacts of copper (Cu) on bacterial community composition and diversity along Cu gradient (20 to 3537 mg·kg^−1^), which had been contaminated with CuSO_4_ for more than 85 years. Their results did not demonstrate any significant correlation between bacterial OTU richness and bioavailable Cu, expressed as the concentration of bioavailable Cu (measured with a biosensor using *Pseudomonas fluorescence*) divided by the concentration of water-extractable Cu (0.006 and 1.90 mg·kg^−1^), although the long-term Cu exposure selected for copper-tolerant soil bacterial communities with changed composition. Brandt *et al.* [70] reported that soil bacterial communities can exhibit structural (terminal restriction fragment length polymorphism (T-RFLP) analysis) and functional (soil respiration) resilience to a five-year Cu exposure (plots amended with 63 g Cu, as CuSO_4_ per m^2^, which, when homogenously distributed over the top 5 cm, would correspond to approx. 900 mg·kg^−1^) to subsequent Cu amendment in soil microcosms (0, 40, 150 or 500 mg·kg^−1^) by developing Cu tolerance without obvious changes in overall community structure. They claimed that the observed increased Cu tolerance to high copper concentrations may involve phenotypic adaptation or selection at the micro-diversity level, e.g., an increase in the copper-resistant strains within each bacterial species. Hong *et al.* [71] studied the diversity and composition of bacterial communities in soils sampled from different iron mining areas polluted with Cd (0.09 to 0.75 mg·kg^−1^), Cr (110 to 324 mg·kg^−1^), Cu (32 to 62 mg·kg^−1^) and Fe (104,000 to 24,000 mg·kg^−1^); although Cr and Cd contamination was rather low, the average concentrations of both metals were still approximately three to four times higher than the average for Chinese soils. Based on Illumina MiSeq sequencing, they showed that metal-impacted soil had a significantly higher bacterial alpha diversity compared with unpolluted soils. Li *et al.* [72] used high-throughput sequencing to study the responses of soil microorganisms under five-year nickel pollution (0.8 to 94 mg·kg^−1^) in two contrasting agricultural soils in China. Despite obvious shifts in the bacterial community composition, no clear trend was observed in the bacterial diversity and abundance across the nickel gradient. To link the microbial community composition and function, Chen *et al.* [73] used pyrosequencing-based comparative phylogenetic and metagenomic profiling of microbial communities originating from six tailings produced by a lead/zinc mine. They showed that the microbial communities at a site with high metal concentrations (Pb: 6811 mg·kg^−1^; Zn: 122,400 mg·kg^−1^; As: 1182 mg·kg^−1^; Cu: 109 mg·kg^−1^) and high pH (6.4) and another one characterized by similarly high metal concentrations (Pb: 6813 mg·kg^−1^; Zn: 9460 mg·kg^−1^; As: 1116 mg·kg^−1^; Cu: 106 mg·kg^−1^), but low pH (2.4) harbored more functional genes related to sulfur oxidation/metal detoxification and tolerating low pH, respectively.

Chodak *et al.* [74] studied the effects of metal pollution on microbial communities (using pyrosequencing) in polluted forest soils. High metal concentrations (Cu: 1353 mg·kg^−1^; Zn: 4792 mg·kg^−1^ and Pb: 1877 mg·kg^−1^) negatively affected the Chao1 diversity index while the structure of the soil microbial communities remained stable. In a DGGE-based study of long-term effects (27 years) of copper contamination and pH, De Boer *et al.* [75] found that microbial community structure reflected the contamination history and stabilized after the shift upon Cu contamination (200 mg·kg^−1^). Using the same approach, Renella *et al.* [76] showed that long-term exposure to Cd (40 mg·kg^−1^) had little effect on soil bacterial diversity. Li *et al.* [77] showed that exposure for five years to copper stress in soils artificially polluted with copper chloride powder (3200 mg Cu·kg^−1^) increased the resistance of soil microbial communities (investigated by T-RFLP) to subsequent copper stress (addition of 57 mg Cu·kg^−1^). However, their results revealed little change in the activity of microorganisms (measured as substrate induced respiration and potential denitrification rate) despite a compositional shift, due to functional redundancy.

In a recent study based on pyrosequencing of the 16S rRNA gene and the internal transcribed spacer (ITS) region of fungal rRNA genes, Bourceret *et al.* [78] reported that in industrial wasteland soils contaminated with Cr (658 to 1033 mg·kg^−1^), Cu (131 to 147 mg·kg^−1^), Zn (1926 to 2416 mg·kg^−1^) and Pb (441 to 610 mg·kg^−1^), bacterial and fungal species richness and diversity remained high, indicating a long-term adaptation of the microbial communities developing towards a diversified and metal-resistant community. Epelde *et al.* [79] found a higher functional diversity compared to uncontaminated soils, when using GeoChip 2.0 to explore microbial communities in Zn (1000 mg·kg^−1^) and Cd (250 mg·kg^−1^) contaminated soils in microcosms. Their results suggest that zinc and cadmium resistance genes increased considerably in polluted soils, indicating that 10 months of exposure are enough for the development of Zn and Cd resistance genes as a result of metal pollution. Studying two metal gradients in Southern Poland, polluted mainly with zinc (up to 4300 mg·kg^−1^) and lead (up to 2900 mg·kg^−1^), Azarbad *et al.* [80] found that functional potential (assessed by Functional Microarray analysis: GeoChip 4.2 and bacterial taxon richness and community composition (shown by Illumina sequencing of 16S rRNA genes) were highly similar in both pollution gradients [80], revealing, at the same time, significant correlation between overall community structure and metal pollution level. The authors [80] reported that adaptations to metal pollution were primarily due to the changes in the frequency of metal resistance genes (such as *arsA*, *pbrT* and *cusF*) and shift in relative abundances of some bacterial groups. Most likely bacterial diversity and community composition may have changed initially, directly after the smelters became active (1967–1970) [50], after which the metal-adapted bacterial communities may have recovered to their original composition and became colonized by new (resistant) populations during the next decades.

Taken together, soils surrounding metal smelters were unpolluted before smelting started and then, over decades, have become highly contaminated. This means that soil microbial communities in such areas probably had a good chance to become adapted. Although the majority of microbial populations may have become extinct after metals reached toxic concentrations, certain community members with higher metal resistance survived and founded new communities. This long-term process should lead to the development of metal tolerant communities after a few decades. It may also contribute to understanding why it is so difficult to compare results from one field study to another as each situation has a different history, not only in terms of soil type and metal mixture but also in terms of speed at which it became polluted, and therefore the rate at which microbial communities could (or could not) adapt to the changing situation.

Although the application of advanced molecular techniques theoretically warrants better and more comprehensive assessment of microbial communities under pollution stress, a number of important issues remain unresolved. In their recent review, addressing the responses of soil bacterial microflora to pesticide exposure, Imfeld and Vuilleumier [58] identified some of these problems. A major limitation of the use of molecular techniques is that bacterial phylotypes that significantly contribute to observed changes in community structure may be difficult to identify precisely due to the high species richness and inter-individual variability of the microbiota. In addition, even if discrimination at the phylotype level can be achieved, the obtained information may lack the functional relevance. For example, Azarbad *et al.* [80], in a study on two long-term metal polluted forest soils, reported that, despite the observed changes in phylogenetic diversity, the functional potential of the communities remained stable across the pollution gradient. Variation in functional potential gene structure, which was not well explained by phylogenic variation, revealed that phylogenetically distant taxa may have similar functions [81]. Many functional traits are closely related to taxonomy, while others are not. If such functional traits are distributed among a few taxa and phylogenetic clades, shifts in the community composition have the potential to strongly alter the associated ecosystem functioning [82]. In contrast, microbial communities can be resilient to changes if functional traits are distributed among various groups of microbes [83]. Traits that are exchanged via mobile elements (like metal resistance genes) are likely to be less related to phylogeny. Functional genes may be exchanged through extensive horizontal transfer of genetic material between species, which may lead to a community gaining metal resistance but otherwise being mostly similar in composition to communities that have not been exposed to metals [75].

It is important to note that, assuming the methodology is sensitive enough to detect changes in the community (which sometimes is not the case), no change in community does not indicate stability but rather shows that the stress of metal exposure is too low to affect the community. As we showed above, some studies reported no correlation between microbial diversity and metals that might have been caused by the low concentration or the relatively low toxicity of the metals (see, for example, [68,84]).

Soil physicochemical conditions can also strongly influence the response of soil microorganisms to metal pollution [21]. The toxicity of metals depends on such natural soil properties as pH, organic matter content and soil texture, with evidence that high clay content promotes bacterial diversity [85,86]. Uptake of metals by microbes can also vary across soil types, and most likely is restricted to the ionized form [7].

As discussed above, microbial communities may vary in their responses to metal pollution in different ecosystems. The likely reasons for the differentiation in tolerance towards metal pollution are: the type and concentrations of the contaminating metals, environmental settings, starting situation, and the pollution history, with the rate at which metal pollution was built-up being probably the crucial factor. Results of the studies reviewed above clearly show the complex nature of the effects of long-term exposure to metals on community adaptation, and suggest that further field studies of metal-polluted soils, with a long-term history of contamination, are needed to elucidate the interactions between metal pollution, soil physicochemical conditions and microbial diversity.

## 4. Mechanisms of Metal Adaptation and Tolerance in Microbial Communities

The application of high-throughput genomic tools, together with more conventional biochemical and physiological community analyses, has provided unique insights into the adaptation strategies used by microorganisms to cope with metal pollution. Long-term metal exposure can result in (1) changes in community function [19,21,87,88]; (2) changes of the community structure and composition [61,62,63,89]; or (3) the development of community tolerance to metals e.g., due to selection of metal-resistant communities [80,90,91]. Pollution-induced community tolerance (PICT) is the concept that has been widely used to describe changes in microbial communities resulting from exposure to contaminants and bringing increased tolerance towards pollutants [92]. The three main mechanisms behind PICT include: (1) physiological adaptation, e.g., adaptation of membrane permeability, or activation of tolerance genes; (2) replacement of sensitive species by more tolerant ones; and (3) adaptive mutation (stress-induced mutagenesis) or resistance by acquisition of metal resistance via horizontal gene transfer. These mechanisms and also other adaptation mechanisms to metals have been described in multiple research articles and reviews and are not covered in detail in this review (see, for example, [93,94,95,96,97]).

Adaptation to metal pollution at the level of microbial community has been reported for several soils contaminated with zinc, copper or cadmium [98,99,100]. The necessity to cope with metals induces, however, an increased energy demand in microbes due to the maintenance costs of the resistance mechanisms. In the following sections, we discuss how energetic costs of maintaining these mechanisms may influence the responses of metal-tolerant communities to a secondary stressor in a “stress-on-stress” situation. To address this issue, we summarize current understanding of the stability of soil microbial communities (resistance and resilience) and discuss the underlying mechanisms that govern microbial stability, focusing on metal pollution as the initial stress, in the face of additional stressors, and further discuss possible factors affecting microbial stability.

## 5. Stability of Microbial Communities (Resistance and Resilience) to Secondary Stressors in Metal-Polluted Environments

**Figure 1:**
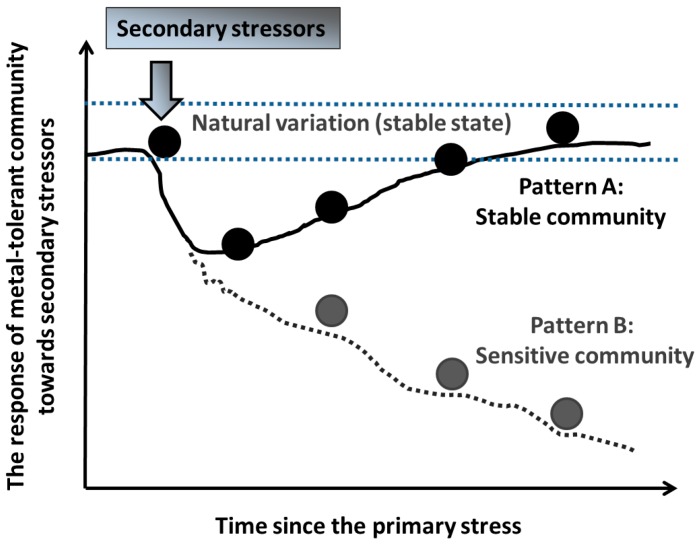
Conceptual figure showing the stability and/or sensitivity of metal-tolerant microbial communities that have been chronically exposed to metals and then face a secondary stressor. **Pattern A: Stable community**—A metal-tolerant community exhibits increased stability and resistance to secondary stressors; **Pattern B: Sensitive community**—Results from energetic trade-offs between resistance to different stressors or negative correlations between the traits determining the ability of microbial communities to withstand different stressors (for explanations, see text).

Several theories/patterns have been proposed regarding the stability of microbial communities living under chronic exposure to metals and facing additional stressors [101]. Using the conceptual model developed by Vinebrooke *et al.* [101], with some modifications, we propose two types of responses (Figure 1). The first pattern A suggests that microbial communities exposed to elevated levels of metal pollution are more stable than those that have never experienced elevated metal concentrations, as they have acquired certain universal physiological adaptations that allow them to maintain their function and structure without major changes in the face of a range of stressors. This may happen when the detoxification of a secondary stressor relies on similar physiological pathways as those acquired/modified to cope with the primary one. For example, resistance to one specific metal may also provide cross-resistance or co-tolerance to other metals (combined resistance to several metals). Indeed, Díaz-Raviña *et al.* [100] showed that bacterial community tolerance (assessed by the thymidine incorporation method) to one metal often led to increased tolerance for other metals. Thus, the co-tolerance increases the resistance to one stressor as a result of an earlier exposure to a similar stressor [34,100,101,102,103]. However, because different tolerance or resistance genes can be located on the same plasmid, a tolerance may also occur to a stressor completely different than the primary one exerting the selection pressure [104,105].

A recent study on microbial communities originating from two gradients with different pollution sources and histories showed that long-term exposure to metal pollution enhanced functional (respiration rates) and structural (assessed by DGGE) stability of soil microbial communities when exposed to additional metal (arsenic) and salt (NaCl) stress. However, the very same communities did not show increased resistance against benzo[a]pyrene and flooding stress applied as secondary stressors [18]. This suggests that soil microbial communities adapted to high metal concentrations are able to better tolerate only certain additional stressors. The mechanism of the observed metal adaptation is probably due to the changes found in the frequency of metal resistance genes (including *arsA*), as compared with communities that experienced lower pollution levels [80]. The authors suggested that the higher frequency of the metal resistance gene in the community previously exposed to high levels of Zn, Pb and Cd was the key factor in the response towards arsenic. In the case of increased tolerance to salt stress in most polluted sites, the authors claimed that it could be due to the effect of the ionic strength in soils polluted by several metals [18].

The second pattern B predicts increased sensitivity of metal-tolerant communities to a second stressor, which results from a trade-off between the traits that determine the ability of microbial communities to withstand each stressor (Figure 1). Local adaptation to metal pollution may cause lower resistance against new stressors, resulting from the cost of adaptation towards metals. Genetic variation between microbial populations is maintained by these trade-offs, resulting in physiological specialization [106,107], and may promote ecological speciation, that is the process by which barriers to gene flow develop between populations as a consequence of ecologically-based divergent selection [108]. Here, the stability of microbial communities is likely to be severely reduced after exposure to a second stressor, if metal stress, as the initial stressor, eliminates certain microbial species and selects resistant ones. Then, the surviving tolerant species on average will have an increased sensitivity to secondary stressors. For example, Müller *et al.* [36] reported that soil bacterial communities that have been exposed to mercury for more than 14 years showed increased functional sensitivity (measured as CO_2_ production) to a subsequent stressor (heat treatment) [36].

## 6. Factors Affecting Microbial Responses to Secondary Stressors

**Figure 2:**
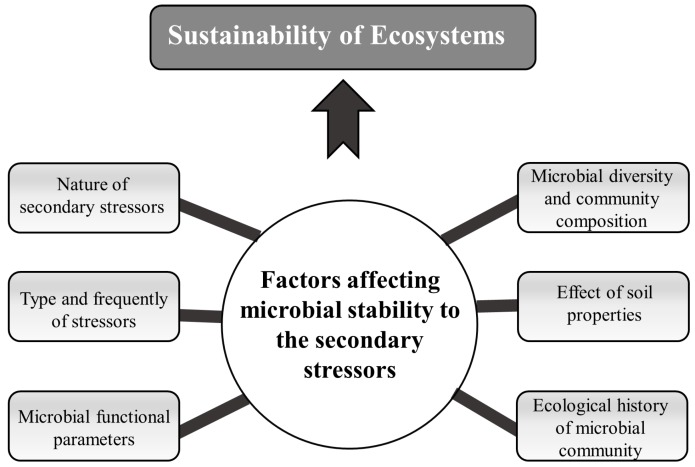
Schematic representation of possible factors affecting the stability of soil microbial communities in historically metal-contaminated fields in the face of secondary stressors.

The following sections focus on our growing understanding of the factors affecting microbial community stability in polluted areas with a long-term history of contamination, including studies not necessarily connected with metal stress but other types of stressors, as evidence is still limited in this field. The main question we address here is the large variability in results of studies on the stability of microbial communities facing secondary (additional) stressors. Although it seems that the most important factors determining stability of microbial communities exposed to environmental stressors have been identified (Figure 2), further studies are needed to evaluate the relative contribution and importance of these factors.

### 6.1. Nature of Secondary Stressors

Because metal contamination is highly persistent, metal-polluted ecosystems are inherently different from those disturbed by natural, short-lived factors, such as, e.g., flooding [109]. Duration and specificity of a stressor are important factors shaping microbial response towards secondary stressors. We argue that the degree to which a community is resistant or resilient depends on the initial microbial community [18,110], the nature and level of the secondary stressor [18,34,110], and historical environmental conditions (that is, long-term exposure to toxic levels of metals) [18,35,110]. Disturbances caused by stressors having a specific mode of action may only alter certain groups of microorganisms, whereas stressors with non-specific modes of action can target a wide range of microorganisms. The effect of a transient disturbance is different from that of long-term disturbance (e.g., metal pollution), since inhibited species usually recover quickly from transient disturbance, so that the community can persist in an equilibrium state (resilience). This has been documented in several studies where some soils showed resistance and/or resilience to transient heat, but not to persistent copper addition [111,112,113,114,115,116].

### 6.2. Knowledge of Diversity, Composition and Function of Microbial Communities before and after Secondary Stressors

Previous “stress-on-stress” research in areas with long-term metal pollution history focused mostly on functional aspects of microbial communities, such as changes in respiration rates [33,35,117] and enzyme activity [31,32], after applying secondary stressors. To enhance our understanding of effects of primary and secondary stressors, both functional and structural responses need to be considered [18]. The underlying mechanisms that determine community structure and metabolic potential play important roles in determining the reaction to the initial and the secondary stressor. For example, using two metal pollution gradients, Azarbad *et al.* [18] showed how the knowledge on microbial diversity, community composition, functional and metabolic potential, prior to applying the secondary stressor, and, after it, helped in understanding the functional and structural responses of metal-tolerant microbial communities in face of four different types of secondary stressors (benzo[*a*]pyrene, salt, arsenic and flooding). The authors stressed that, without this knowledge, it would be difficult to pinpoint the factors determining the response of the community after the secondary stressors. We argue that knowledge about factors affecting the structure and the function of microbial communities is pivotal when attempting to predict the impact of secondary stressors on the stability of soil microbial communities.

A number of studies published over the past decade highlighted the role of microbial diversity for ecosystem functioning [118,119,120], including potential consequences of reduced diversity to the stability of microbial communities [121,122]. In ecosystems, the diversity of functional groups undergoing change, within-species genetic diversity, and the diversity of species in functional groups seem to be critical components of the resilience of ecosystem functions [123,124]. Therefore, the knowledge on functional communities, that is, assemblages of populations sharing certain features and functional processes (e.g., nitrification and denitrification), may help in estimating specific microbial responses to metal stress (as initial stress) and further to the secondary stressors.

It has been shown that microbial diversity is especially important for stability of narrow-scale functions, such as those associated with nitrification and denitrification [11,125]. General functions, e.g., organic matter decomposition and soil respiration, are much more resilient due to the functional redundancy [122]. The effect of environmental stressors on the response of a soil microbial community to additional stress may depend on the groups within the community that are tolerant to that particular stressor [112,113,126,127]. Another important point to note is that functional stability of microbial communities is often determined by specific components within communities [113,128]. Hence, the overall community structure and composition, rather than diversity itself, can be appropriate traits(s) to assess the effects of environmental stressors on the functioning of soil microbial communities [129]. However, two key questions need to be answered: (1) which microbial components contribute most to functional stability of microbial communities; and (2) which abundant and rare taxa show similar or different responses toward environmental stressors.

### 6.3. Microbial Functional Parameters

Soil microorganisms perform many important functions, and the dynamics of different functional groups following the exposure to stressors can be different [15]. Soil microbial activity [130,131], organic matter decomposition [111,112], denitrification and nitrate oxidation [122] have been used as soil quality indicators in studies on the stability of soil microbial communities to additional stress [132]. Functional resilience, defined as recovery of a soil microbial process, is often observed in soil microbial communities, but the degree to which resilience is possible depends on the functions measured and the type and dose of the applied stress [13,112,133,134].

Contrasting results were observed when different microbial parameters were compared in the face of additional stressors. In general, previous studies indicated that the effect of stressors on the functional stability of the soil microbial community depends on the level of specificity of the function measured. It has been well documented that soil respiration is less sensitive to stressors than more specific parameters, such as enzyme activity. This is due to the fact that a wide range of organisms contributes to respiration, while only a small fraction of the total microbial community is responsible for nitrification [111,112,135]. Kandeler *et al.* [136] reported that enzyme activities related to the cycling of N, P and S were less resistant to increasing metal contamination than those involved in the mineralization of organic matter. Using copper-polluted (800 mg·kg^−1^) and unpolluted soils, Deng *et al.* [103] studied the effect of initial copper pollution on the stability of microbial communities after exposure to additional metal stress (Cu at concentrations 400 and 800 mg·kg^−1^). They showed that the number of copies of 16S rRNA genes, microbial biomass carbon (C_mic_), and substrate-induced respiration (SIR) (considered as general parameters) were more stable to additional metal spiking (CuSO_4_) than the number of copies of *amoA* gene and potential ammonia oxidation rate (classified as specific parameters).

### 6.4. Field vs. Laboratory Experiments

Reports focusing on the responses of microbial communities towards a secondary stressor can be divided into two types. The first type consists of field observations after long-term (at least 10 years long) pollution with such metals as copper [31,33,35,137], zinc [117], or mercury [36]. The second type of stability studies includes a large body of laboratory-based research that typically uses microcosms with only one or a few environmental factors in short-term experiments. Care should be taken when comparing long-term studies with short-term laboratory experiments. Of course, a downside of all microcosm experiments is that they do not necessarily reflect natural environmental conditions and, thus, field trials must be conducted to confirm the results of laboratory experiments [138]. Under field conditions, where microbial communities are exposed to long-term history of contamination, the observed shifts in microbial community structure do not necessarily correspond to the patterns observed right after a single pollution load, as is generally the case in controlled laboratory experiments [139]. In addition, time taken to build-up concentration in the field is of importance, and so is the difference in metal form applied in the lab and received in the field.

### 6.5. Effect of Soil Properties

Previous studies have shown that the functional stability of microbial communities changes with soil properties [18,116]. Soil can provide shelter for microorganisms and act as a buffer against the stressors [103]. Microbial diversity may decline in response to a stressor, as stressors favor those microbes that are best adapted to cope with the given stress. On the other hand, some microbial community properties, including diversity, may increase in response to stressors thanks to the enhancement of spatial heterogeneity of the soil parameters (that is, natural variation of soil properties from one point to another in space), promoting co-existence of a higher number of species [140,141]. Soil pH is one the primary factors affecting metal bioavailability in soils, but other soil physicochemical conditions, such as cation exchange capacity and organic matter contents, have also significant influence on the bioavailability of toxic compounds to microorganisms [9,142,143]. Therefore, soil physicochemical conditions may have a significant effect on the stability of microbial communities. For example, soils with high contents of organic matter or clay can buffer effects of certain stressors [144,145]. This was confirmed by a survey of 26 different soils from arable land, grassland, moorland and woodland across Scotland, which indicated that resistance of soil microbial communities to copper addition was related to soil organic matter content and soil pH [114].

## 7. Conclusions

To improve our understanding of the combined impact of metal pollution and secondary stressors on soil microbial communities, it is crucial to learn which microbial groups are selected in communities under strong pressure by metal stress and how associated costs of tolerance affect their responses in the face of additional stressors. There are several issues that still need to be addressed in order to evaluate the effect of metal pollution and secondary stressors on microbial communities in metal impacted soils. In this review, we showed that historical environmental conditions need to be considered when attempting to predict stability of microbial communities towards secondary stressors. Furthermore, microbial communities selected for metal resistance, in some cases, did not show a loss of their capabilities to cope with additional stress (due to acquired co-tolerance). Indeed, for some stress factors, the tolerance and resistance of soil microbial communities appeared elevated rather than decreased. Therefore, we may expect that if a microbial population has adapted genetically to long-term metal contamination, it may have higher tolerance and/or a higher ability to deal with additional environmental stressors. This is an important aspect for predicting responses of metal-tolerant communities to future environmental impacts, such as climate change, in areas with a long-term history of contamination.

In historically contaminated areas, functional redundancy buffers selective pollution effects, allowing soil microbial communities to perform their functional roles at relatively unchanged levels, despite the observed shifts in community structure. It may be, thus, difficult to evaluate ecological risks to such ecosystems based solely on measuring functional performance of their communities. In order to identify areas that are at high risk of losing their functionality due to pollution, microbial communities from such areas should be exposed experimentally to secondary stressors in order to see whether they show any reduction in functional capabilities. Such “stress-on-stress” studies should be designed with the following issues in mind:

(1) The application of new DNA-based methodologies, allowing culture-independent assessment of microbial communities, has greatly enhanced our insights into the composition of these communities and their functional capacities.
(2) There is increasing support that the historical environmental conditions and disturbance regimes are primary factors to influence microbial community stability towards secondary stressors. We argue that knowledge of factors controlling the composition and function of microbial communities is pivotal when attempting to predict the impact of secondary stressors on the stability of soil microbial communities. The potential influence of past events (such as metal pollution) on the development of community co-tolerance highlights the importance of historical factors in predicting the response of microbial communities to additional stressors.
(3) Long-term “stress-on-stress” studies with single metals or defined mixtures may provide an integrated insight into the complex responses of soil microbial communities to secondary stressors at polluted sites. Following severe or long-term stress exposure, microbial community may not be able to recover within a short-term period.
(4) The effect of soil physicochemical properties is important. The effects of metal pollution on microbial community structure is often confounded by natural soil properties such as pH and organic matter content.
(5) Detailed knowledge on the functional and compositional properties of the microbial community prior to applying additional stressors may help to evaluate and interpret the microbial response to these stressors.
(6) In contrast to DGGE and PLFA, far less attention has been given to sequence data to examine the direct or indirect interactions between microbial taxa in the face of stressors. We recommend that future “stress-on-stress” studies should investigate microbial communities under environmentally stressful conditions, using metagenomics and meta-transcriptomics together with carefully designed disturbance experiments and using different types of stressors. We expect that such studies will provide a deeper insight and clearer picture on whether predictable species assemblages occur following the combination of metal stress with other environmental stressors.

## Figures and Tables

**Table 1:** Summary of definitions used in this article.

Terms	Definitions	References
Adaptation	Increase of genetically encoded traits that enhance the fitness of their bearers.	[53]
Community composition	The richness, relative abundance, and phylogenetic structure of taxa in an assemblage.	[11]
Community structure	Taxonomic composition of a microbial community; can also refer to the spatiotemporal distribution of taxa.	[53]
Costs of tolerance	Any deprivation of fitness-related traits that is a consequence of altered resource allocation involved with adaptation to stress.	[29]
Ecological history	Ecological and evolutionary events that have occurred at some point in the past, such as dispersal limitation, drift, priority effects, or selection by past environmental conditions.	[54]
Functional redundancy	The ability of one microbial taxon to carry out a process at the same rate as another taxon under the same environmental conditions.	[11]
Functional stability	The ability of a microbial community to minimize dynamic fluctuations of a function (such as respiration rate, enzyme activity or functional potential gene structure) and to defy changes in the community after a disturbance.	[13]
Resilience	The capacity of a community under stress to persist and maintain or recover their original or new stable state in terms of composition and function.	[55,56,57]
Resistance	The degree to which a community withstands changes in the face of disturbance. The ability of a community to maintain population structure and function under a toxicity stress.	[11,55,56,57]
Stability	The tendency of a community to return to a stable condition after stress; includes the components of resistance and resilience.	[54]
Stress	A deviation from optimal conditions that leads to a reduced growth rate or a cellular damage in result of environmental or internal changes.	[53]
Tolerance	The ability of a community to withstand toxic insults inflicted by pollutants on the ecosystem, and survive under the resulting conditions. Tolerance merges aspects of physiological adaptation and resistance of microbial populations in a single concept.	[58]
Trade-offs	Negative correlation between two life-history (or other) traits in such a way that an increase of one trait imposes a cost to another.	[59]
